# Exploring the mechanism of action of Xuanfei Baidu granule (XFBD) in the treatment of COVID-19 based on molecular docking and molecular dynamics

**DOI:** 10.3389/fcimb.2022.965273

**Published:** 2022-08-10

**Authors:** Li Xiong, Junfeng Cao, Xingyu Yang, Shengyan Chen, Mei Wu, Chaochao Wang, Hengxiang Xu, Yijun Chen, Ruijiao Zhang, Xiaosong Hu, Tian Chen, Jing Tang, Qin Deng, Dong Li, Zheng Yang, Guibao Xiao, Xiao Zhang

**Affiliations:** ^1^ Clinical Medicine, Chengdu Medical College, Chengdu, China; ^2^ Chengdu Medical College of Basic Medical Sciences, Chengdu, China; ^3^ Department of Infectious Diseases, First People’s Hospital of Ziyang, Ziyang, China

**Keywords:** COVID-19, Xuanfei Baidu granule, bioinformatics analysis, molecular docking, molecular dynamics

## Abstract

**Purpose:**

The Corona Virus Disease 2019 (COVID-19) pandemic has become a challenge of world. The latest research has proved that *Xuanfei Baidu granule* (XFBD) significantly improved patient’s clinical symptoms, the compound drug improves immunity by increasing the number of white blood cells and lymphocytes, and exerts anti-inflammatory effects. However, the analysis of the effective monomer components of XFBD and its mechanism of action in the treatment of COVID-19 is currently lacking. Therefore, this study used computer simulation to study the effective monomer components of XFBD and its therapeutic mechanism.

**Methods:**

We screened out the key active ingredients in XFBD through TCMSP database. Besides GeneCards database was used to search disease gene targets and screen intersection gene targets. The intersection gene targets were analyzed by GO and KEGG. The disease-core gene target-drug network was analyzed and molecular docking was used for verification. Molecular dynamics simulation verification was carried out to combine the active ingredient and the target with a stable combination. The supercomputer platform was used to measure and analyze the number of hydrogen bonds, the binding free energy, the stability of protein target at the residue level, the solvent accessible surface area, and the radius of gyration.

**Results:**

XFBD had 1308 gene targets, COVID-19 had 4600 gene targets, the intersection gene targets were 548. GO and KEGG analysis showed that XFBD played a vital role by the signaling pathways of immune response and inflammation. Molecular docking showed that I-SPD, Pachypodol and Vestitol in XFBD played a role in treating COVID-19 by acting on NLRP3, CSF2, and relieve the clinical symptoms of SARS-CoV-2 infection. Molecular dynamics was used to prove the binding stability of active ingredients and protein targets, CSF2/I-SPD combination has the strongest binding energy.

**Conclusion:**

For the first time, it was found that the important active chemical components in XFBD, such as I-SPD, Pachypodol and Vestitol, reduce inflammatory response and apoptosis by inhibiting the activation of NLRP3, and reduce the production of inflammatory factors and chemotaxis of inflammatory cells by inhibiting the activation of CSF2. Therefore, XFBD can effectively alleviate the clinical symptoms of COVID-19 through NLRP3 and CSF2.

## Graphical Abstract

The mechanisms analysis of *Xuanfei Baidu Granules (XFBD)* in the treatment of COVID-19.

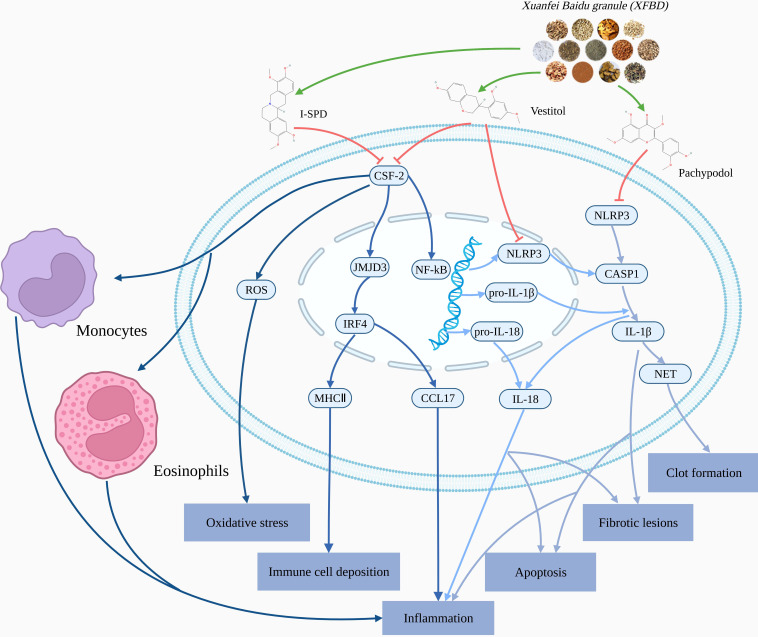

## Introduction

Coronavirus disease 2019 (COVID-19) is a highly infectious disease caused by severe acute respiratory syndrome coronavirus 2 (SARS-CoV-2) (Lai et al., 2020). Since the outbreak of COVID-19, it has been characterized by strong infectivity, long treatment time after infection, and high mortality of patients with severe illness (Fogarty H, et al., 2020; Rowan NJ, et al., 2020; Goldman DT, et al., 2021). The latest clinical research findings that the main physiological and pathological feature of severe COVID-19 is “cytokine storm”, also known as inflammatory storm (Ouédraogo et al., 2020). It is an immune response produced by a positive feedback loop between cytokines and immune cells, and it is also the state which the body’s immune system has evolved from “self-protection” to “over-protection” (Bellanti and Settipane, 2020). Therefore, the outbreak of inflammation is an important pathological factor leading to the aggravation and even death of patients with respiratory damage caused by COVID-19 (Liu et al., 2021), however, there is no clear antiviral therapy for COVID-19 in the clinic (Panyod et al., 2020). The latest clinical trials show that traditional Chinese medicine has a significant effect on viral pneumonia. Clinical studies have shown that XFBD combined with conventional drugs can significantly improve clinical symptoms such as fever, cough, fatigue, loss of appetite, etc. XFBD treatment can increase the number of white blood cells and lymphocytes to improve immunity, while significantly reducing C-reactive protein and erythrocyte sedimentation rate to play an anti-inflammatory effect (Xiong et al., 2020; Zhao et al., 2021a). Meta-analysis demonstrated that XFBD alleviated clinical symptoms in most patients with mild or moderate COVID-19, and reduced the transition of mild patients to severe disease (Runfeng et al., 2020; Wang et al., 2022).At present, the symptomatic treatment of COVID-19 with integrated traditional Chinese and Western medicine has been clinically applied in China, and good therapeutic effects have been achieved.

Currently, the National Health Commission of China recommends the traditional Chinese medicine compound *Xuanfei Baidu Granule* (XFBD) for the clinical treatment of COVID-19 (Xie, 2020).

*Xuanfei Baidu granule* (XFBD) consists of 13 Chinese materia herbs: bitter almond, atractylodes, artemisia annua, patchouli, polygonum cuspidatum, verbena, reed root, ephedra, coix seed, exocarpium, licorice, semen lepidii, and gypsum (Zhao et al., 2021a). XFBD is a traditional Chinese medicine compound for the treatment of anti-epidemic, which is designed for the pathological characteristics of wet toxin (Xie, 2020). XFBD has the effects of inhibiting viral infections, promoting the absorption of lung inflammation, and reducing inflammatory factors.

A large number of clinical studies have shown that *Xuanfei Baidu granule* (XFBD) can effectively relieve the clinical symptoms of COVID-19 patients (Li et al., 2021a). The latest clinical study found that XFBD combined with conventional drugs significantly improved the clinical symptoms of COVID-19 patients, increased the number of white blood cells and lymphocytes, and decreased C-reactive protein and erythrocyte sedimentation rate. This result suggested that XFBD had a potential immunomodulatory role in the treatment of COVID-19 (Xiong et al., 2020).

However, there is currently a lack of more in-depth and systematic research on *Xuanfei Baidu granule* (XFBD) in the treatment of COVID-19. And XFBD is a traditional Chinese medicine compound, its complex components also hinder the related research in the treatment of COVID-19. Molecular dynamics can comprehensively and systematically simulate the interaction and binding stability between small molecule monomers and protein targets with the help of powerful computing power.

Molecular dynamics (MD) is an interdisciplinary subject based on the knowledge of physics, chemistry, life science, materials and other disciplines. It uses large computer clusters (or even supercomputers) as the carrier, it aims to obtain data such as microstructure, physical and chemical properties, and performance characterization parameters of materials by calculation (Santos et al., 2019). It is a supplement and in-depth excavation of the traditional materials discipline mainly based on experiments. Through the data obtained by calculation, the mechanism behind the experiment is researched and analyzed at multiple levels from the microscopic, mesoscopic and macroscopic scales. So that it is not only limited to “qualitative”, but can rise to the theoretical height of “quantitative” (Anuar et al., 2021). It analyzes the behavioral law of molecular motion by solving the potential function of intermolecular interaction and the equation of motion, simulates the dynamic evolution process of the system, and provides microscopic quantities (such as: the coordinates and velocity of molecules, etc.) and macroscopic observable quantities (such as: the relationship between the temperature, pressure, heat capacity of the system, etc.) (Sivakumar et al., 2020), so as to study the equilibrium properties and mechanical properties of the composite system, it is an effective research method to study the properties of drugs and protein stability. Firstly, molecular dynamics solves the equation of motion for a many body system composed of atomic nuclei and electrons. Secondly, molecular dynamics can not only directly simulate the macroscopic evolution characteristics of matter, but also obtain calculation results that are consistent with or similar to the experimental results. Finally, molecular dynamics can give the microscopic evolution process of the system from the atomic level, and intuitively show the mechanism and law of the experimental phenomenon. Therefore, molecular dynamics can provide a clear picture of the microstructure, particle motion and their relationship with macroscopic properties. Molecular dynamics can also make our research more efficient, more economical, and more predictable.

This study used bioinformatics to screen out potential effective monomers from *Xuanfei Baidu granule* (XFBD). The core intersection targets of XFBD and COVID-19 were screened by GeneCards database. PPI, GO and KEGG were used to analyze the potential associations between gene targets to explore the mechanisms of action and potential pathways. Molecular system movement was used to their simulate the result of calculating interrelationships from the cellular level to the chemical group level. Molecular docking was used to determine the affinity of monomeric compounds and protein targets, molecular dynamics was used to simulate the stability of bound complexes. The research on the mechanism of XFBD in the treatment of COVID-19 will promote its clinical application, lay a solid foundation for related research and promote further research.

## Material and methods

### Identification and screening of active compounds

Traditional Chinese Medicine Systems Pharmacology Database (TCMSP, http://tcmspw.com/) was used to screen and analyse all compounds of the thirteen Chinese medicinal herbs in *Xuanfei Baidu granule* (XFBD) (Daina et al., 2019). Compounds of XFBD are screened according to two key parameters, namely oral bioavailability (OB) and drug similarity (DL), in the assessment categories of absorption, distribution, metabolism and excretion. OB was defined as the degree to which active ingredients are used by the body (Ru et al., 2014). OB largely determines the effect of the compound on the disease, DL is used to screen and refine candidate compounds early in drug development. In this study, the active compounds in XFBD were selected according to the criterion of OB≥30% and DL≥0.18 (Xu et al., 2012).

### The intersection of disease and drug gene targets

We used the GeneCards (https://genecards.weizmann.ac.il/v3/), “COVID-19” and “SAR-Cov-2” were uesd to be the key words to obtain the disease gene targets, and COVID-19-related genes were screened from genecard with relevance score≥5 as the threshold, relevance score is a comprehensive evaluation of the association between genes and research diseases. We also imported the 13 Chinese materia herbs of *Xuanfei Baidu granule* (XFBD) into genecards to obtain drug gene targets. The drug gene targets and the disease gene targets were combined through the venny website to obtain intersection gene targets.

### Xuanfei Baidu granule treatment of COVID-19 interaction protein targets (Protein-Protein Interaction) network building

The STRING database was used to analyze the protein-protein interaction (PPI) of *Xuanfei Baidu granule* (XFBD) in the treatment of COVID-19. STRING database covers the majority of known human protein–protein interaction information (Szklarczyk et al., 2019). In order to further clarify the interaction between potential protein targets, all potential therapeutic protein targets of XFBD on COVID-19 were imported into Cytoscape 3.7.1 to analyze (Shannon et al., 2003), we defined the protein type as “Homo sapiens”, and obtained relevant information on protein interactions by STRING database. Finally, the network topology parameters were analyzed by Cytoscape 3.7.1, and the hub protein targets were screened out according to the criterion that the node degree value and the betweenness center value were greater than the average value.

### The gene target enrichment analysis

The interaction gene targets were used in DAVID database for gene ontology (GO) functional annotation and Kyoto Encyclopedia of Genes and Genomes (KEGG) enrichment analysis. We obtained the molecular function (MF), cellular component (CC) and related biological process (BP) of the gene targets through GO enrichment. The disease-related targets obtained from screening were input into the DAVID database by entering the list of target gene names and selecting the species as “homo sapiens” (Huang Da et al., 2009). In this study, KEGG pathway enrichment analysis was performed on the relevant signaling pathways involved in the target, and gene target screening was performed under the condition of *p*<0.05. The main biological processes and signaling pathways of Xuanfei Baidu Granules (XFBD) on COVID-19 were analyzed. This study visualized the results of GO enrichment and KEGG enrichment by the Omicshare Tools platform (Cao et al., 2022).

### Network diagram of “ Disease-core target gene-drug “

Cytoscape 3. 7. 1 network map software was used to construct a disease-core target gene-drug network and conduct topological analysis. The core gene targets can be screened based on the node degree value greater than two times the median (Cao et al., 2022).

### Component target molecular docking and validation of the docking protocol

Molecular docking was used to study the molecular affinity of *Xuanfei Baidu granule* (XFBD) small-molecule potent antiviral compounds with COVID-19 protein targets. The protein crystal structure used for docking was downloaded from the PDB database, and the 3D structure of the small molecule was downloaded from the PUBCHEM database, and energy minimization was performed under the MMFF94 force field. In this study, AutoDock Vina 1.1.2 software was used for molecular docking work. Before docking, PyMol 2.5 was used to process all receptor proteins, including removal of water molecules, salt ions and small molecules (Kim et al., 2016). Then set up the docking box, use the PyMol plugin center of mass.py to define the center of the docking box based on the position of the crystal ligand, and set the box side length to 22.5 angstroms. In addition, ADFRsuite 1.0 was used to convert all processed small molecules and receptor proteins into the PDBQT format necessary for docking with AutoDock Vina 1.1.2. When docking, the exhaustiveness of the global search is set to 32, and the rest of the parameters remain the default settings. The output highest scoring docked conformation was considered to be the binding conformation for subsequent molecular dynamics simulations (Kim et al., 2016). The study used the original crystal ligand of the protein target as a positive reference, and we analyzed and compared the binding posture of the original crystal ligand and protein, the chemical bond length and the chemical bond angle by re-docking the original crystal ligand and protein. Finally, the consistency of the binding mode can indicate the correctness of the molecular docking protocol (Cao et al., 2022).

### Molecule dynamics

The highest scoring conformations determined by molecular docking analysis were further validated by running 50ns molecular dynamics simulations. Molecular dynamics (MD) simulation is a comprehensive set of molecular simulation methods combining physics, mathematics and chemistry. This method mainly relies on Newtonian mechanics to simulate the motion of molecular systems, we calculate macroscopic properties such as thermodynamic quantities of a system by taking samples from an ensemble of different states of a molecular system.

In this study, all-atom molecular dynamics simulations were performed based on the small molecule and protein complexes obtained from the molecular docking results as the initial structure, and the simulations were performed using AMBER 18 software (Maier et al., 2015). The charge of the small molecule was calculated in advance by the antechamber module and the Hartree–Fock (HF) SCF/6-31G* of the gaussian 09 software before the simulation. Afterwards, small molecules and proteins were described using the GAFF2 small molecule force field and the ff14SB protein force field, respectively. Each system used the LEaP module to add hydrogen atoms to the system, added a truncated octahedral TIP3P solvent box at a distance of 10 Å, and added Na+/Cl- to the system to balance the system charge (Harrach and Drossel, 2014). Finally, the simulated topology and parameter files were exported.

Ligands were parameterized using a generic amber force field (GAFF) using a combination of AmberTools18 and ACPYPE 51 protocols (Wang et al., 2006). After the initial addition of hydrogen atoms to each system, the system uses the steepest descent algorithm for vacuum minimization. Solvent was then added and the system ions were equilibrated using counter ions (Na+/Cl-). The proteins were all energy minimized using the steepest descent method and the conjugate gradient method. This was followed by an NVT and NPT ensemble (1000 ps, dt of 2 fs) and an MD run (100 ns, dt of 2 fs) at 298 K temperature and 1 bar pressure using the skip integrator algorithm. The coordinates and energy of the system are saved every 10 ps. Finally, 50ns production simulations were carried out for each system under periodic boundary conditions. For all simulations, the van der Waals force (vdw) cutoff and short-range electrostatic interactions were set to 10 Å. The Particle-Mesh-Ewald (PME) method is used to evaluate long-range electrostatic interactions. Molecular dynamics simulation trajectories include protein-ligand complex root mean square deviation (RMSD), root mean square fluctuation (RMSF), radius of gyration and solvent accessible surface area (SASA).

### MMGBSA binding free energy calculation

The binding free energy was investigated using the MM-PBSA method, and the conformational stability was studied in detail. The binding free energies between proteins and ligands for all systems were calculated by the MM/GBSA method (Hou et al., 2011). The molecule dynamics trajectory of 50 ns was used for calculation, and the specific formula is as follows:


$$\begin{array}{c} \text{Δ}{\text{G}}_{\text{bind}}=\text{Δ}{\text{G}}_{\text{complex}}-\left(\text{Δ}{\text{G}}_{\text{receptor}}\text{+Δ}{\text{G}}_{\text{ligand}}\right)\\ =\text{Δ}{\text{E}}_{\text{internal}}+\text{Δ}{\text{E}}_{\text{VDW}}+\text{Δ}{\text{E}}_{\text{elec}}+\text{Δ}{\text{G}}_{\text{GB}}\text{+Δ}{\text{G}}_{\text{SA}} \end{array}$$

In the formula, Einternal represents internal energy, EVDW represents van der Waals interaction and Eelec represents electrostatic interaction. The internal energy includes bond energy (Ebond), angular energy (Eangle) and torsional energy (Etorsion); GGB and GGA are collectively referred to as solvation free energy, where GGB is the polar solvation free energy and GGA is the non-polar solvation free energy. For this paper, the GB model developed by Nguyen was used for calculation (igb = 2). The non-polar solvation free energy (GSA) was calculated based on the product of surface tension (γ) and solvent accessible surface area (SA), GSA = 0.0072 × SASA15. The entropy change is ignored in this study due to high computational resource consumption and low precision (Cao et al., 2022).

## Results

### Identification of potentially active compounds in *Xuanfei Baidu granule*

In total, 178 potential compounds in *Xuanfei Baidu granule* (XFBD) were retrieved from the TCMSP database with the criteria of DL≥0.18 and OB≥30%, by further improving the OB score (OB≥74%), five core active compounds in XFBD were screened out, shown in **Table 1**.

**Table 1:** The core active compounds in *Xuanfei Baidu Granules* (XFBD) Binding free energies and energy components.

MOL_ID	Molecule Name	OB	MW	Alogp	Caco2	BBB	DL
MOL013287	Physovenine	106.219	262.34	2.08	0.50	0.20	0.18
MOL012922	I-SPD	87.34	327.41	3.09	0.75	0.20	0.54
MOL007207	Machiline	79.64	285.37	2.82	0.78	0.08	0.23
MOL005890	pachypodol	75.06	356.40	2.99	0.83	0.11	0.39
MOL000500	Vestitol	74.65	272.32	3.14	0.85	0.29	0.20

OB, oral bioavailability.

MW, molecular weight.

BBB, blood brain barrier.

DL, drug similarity.

### Obtained common gene targets by intersection

**Figure 1:**
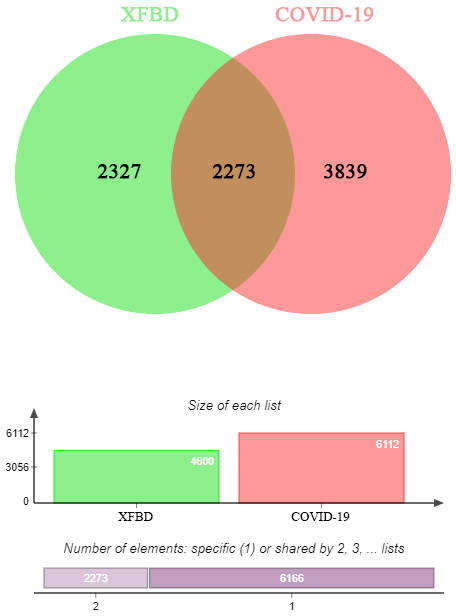
Intersection targets-active ingredient networks. Targets of the intersection of *Xuanfei Baidu granule* (XFBD) and COVID-19.

We obtained 1308 *Xuanfei Baidu granule* (XFBD) gene targets and 4600 COVID-19 gene targets. A total of 548 intersection gene targets were processed by Venny, shown in **Figure 1**.

### Core intersection target screening and PPI network diagram

**Figure 2:**
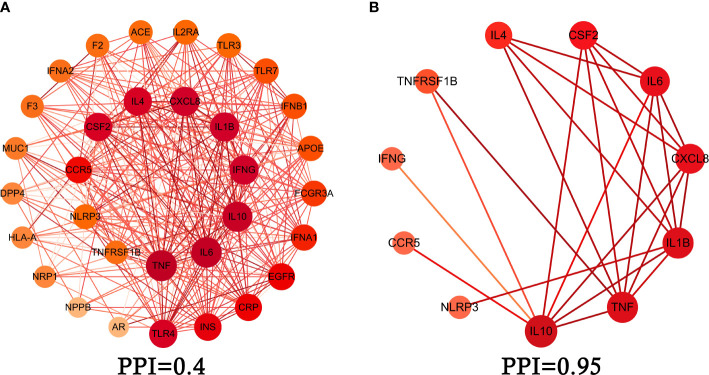
Protein-protein interaction (PPI) network. **(A)** PPI network of protein target, **(B)** PPI network of core protein target (confidence>0.95).

We obtained intersection genes targets of relevance score through GeneCards, relevance score≥5 which were considered as a core intersection gene target, through STRING database analysis of 33 mapping of the core intersection gene targets of COVID-19 and XFBD, the study constructed the PPI network interaction map of the target protein of XFBD in the treatment of COVID-19, shown in **Figure 2A**. 11 core genes (such as CSF2, IFNG, NLRP3, etc.) were obtained by setting the interaction score (confidence degree>0.95), and the study used the 11 core gene targets to reconstruct the core PPI network, shown in **Figure 2B**.

### GO and KEGG enrichment analysis

**Figure 3:**
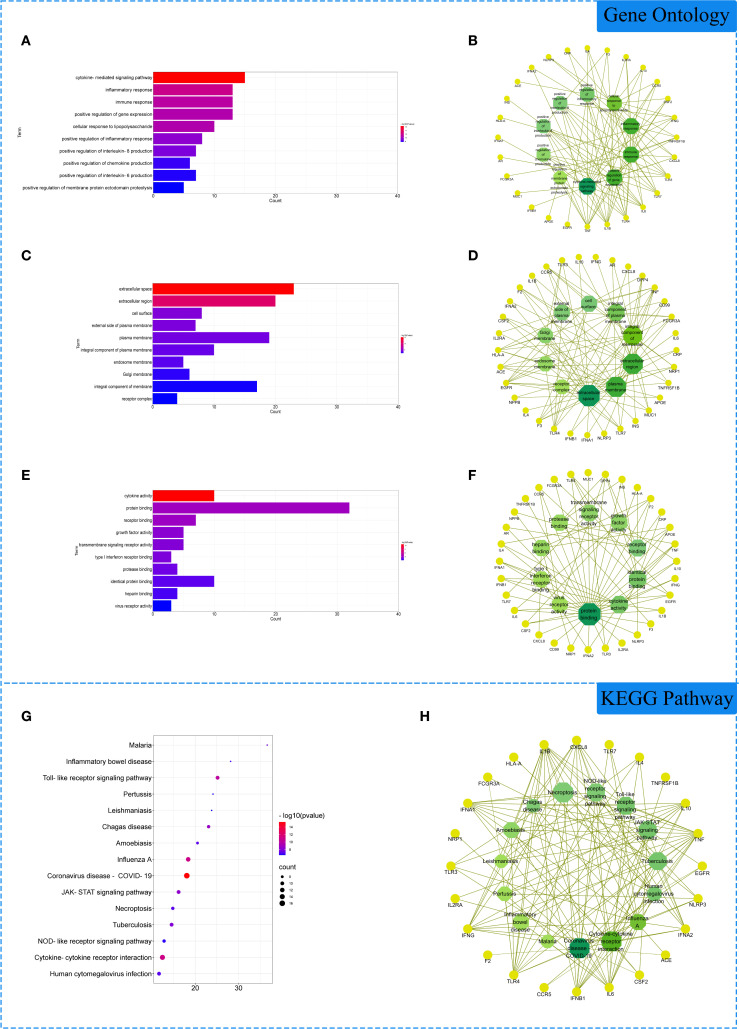
Gene Ontology (GO) and Kyoto Encyclopedia of Genes and Genomes (KEGG) analysis of related genes. **(A)** The top 10 terms in biological processes (BP) were greatly enriched. **(B)** The subnetwork displayed the first 10 BP terms and related genes. **(C)** The top 10 terms in cellular components **(CC)** were greatly enriched. **(D)** The subnetwork displayed the first 10 CC terms and related genes. **(E)** The top 10 terms in molecular function (MF) were greatly enriched. **(F)** The subnetwork displayed the first 10 MF terms and related genes. **(G)** The first 15 KEGG pathways were showed. **(H)** the subnetworks displayed the first 15 KEGG pathways and related.

The 33 intersection gene targets were imported into the DAVID database for enrichment analysis. Under the condition of *p*<0.05, the GO enrichment analysis yielded a total of 277 GO entries, including 239 BP entries, 23 CC entries, and 15 MF entries. According to the number of targets contained, the top 10 BP, CC and MF compressions were screened. The results showed that in biological processes, biological processes were highly correlated with inflammation and viral replication, mainly involving the cytokine-mediated signaling pathway, inflammatory response, and immune response. Among cell components, extracellular space, extracellular region and cell surface account for a relatively large amount. In molecular functions, cytokine activity, protein binding and receptor binding are relatively high, shown in **Figures 3A–F**. KEGG pathway analysis yielded 72 pathways with *p*<0.05. According to the number of targets contained, the first 15 pathways were screened. The results showed that the enriched pathways involved multiple pathways related to inflammation and immune response, mainly coronavirus disease COVID-19, influenza A, cytokine-cytokine receptor interaction and other signaling pathways, shown in **Figures 3G, H**.

### Disease-core gene target-drug network

**Figure 4:**
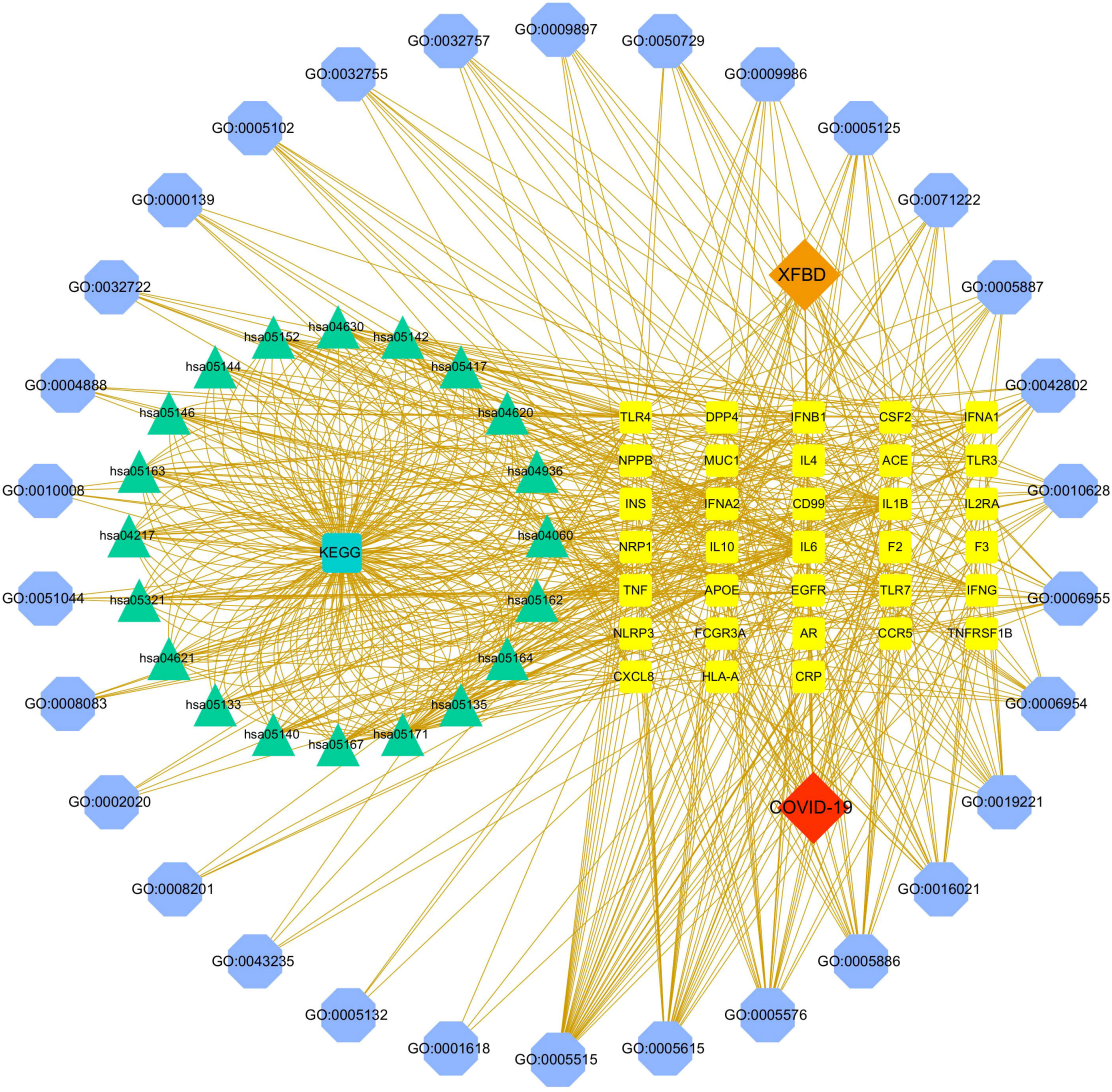
Disease-core gene target-drug network. Square nodes represent gene targets, triangular nodes represent signaling pathways (KEGG), and octagonal nodes represent gene ontology (GO) of related genes.

The disease-core gene target-drug network was constructed to show the main signal pathway and biological process of *Xuanfei Baidu granule* (XFBD) in the treatment of COVID-19, shown in **Figure 4**.

### Molecular docking

**Figure 5:**
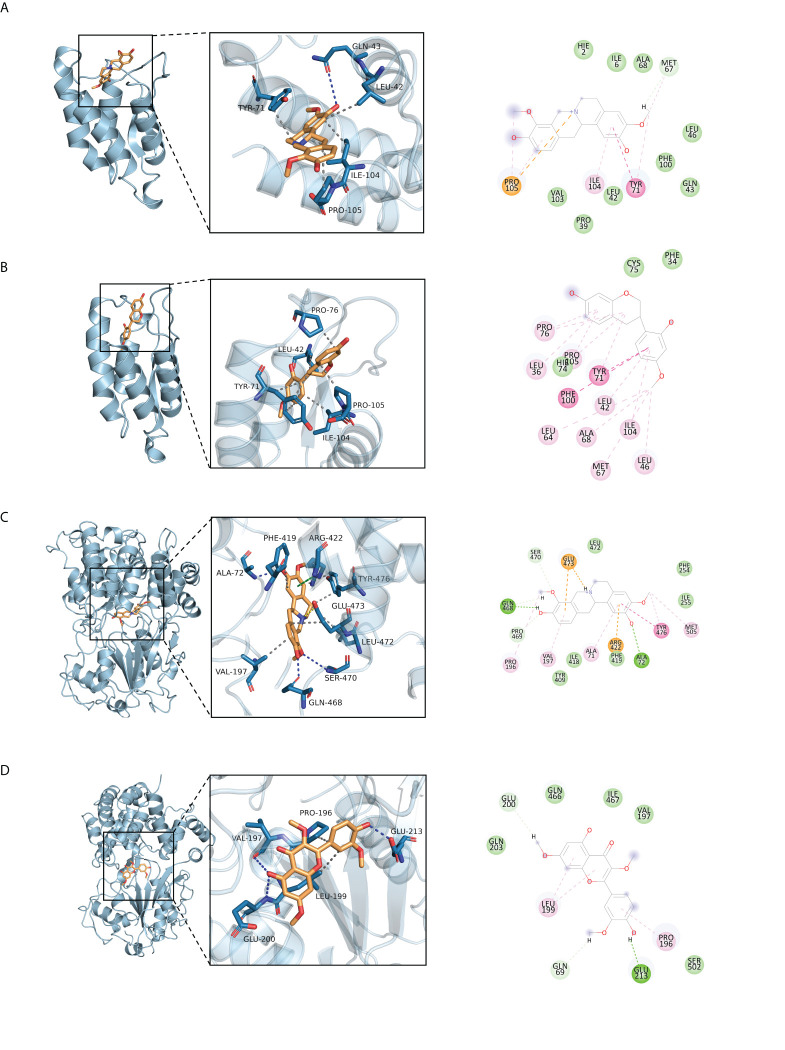
Molecular docking of active ingredients and core targets. **(A)** CSF2/I-SPD, **(B)** CSF2/Vestitol, **(C)** NLRP3/I-SPD, **(D)** NLRP3/Pachypodol.

**Figure 6:**
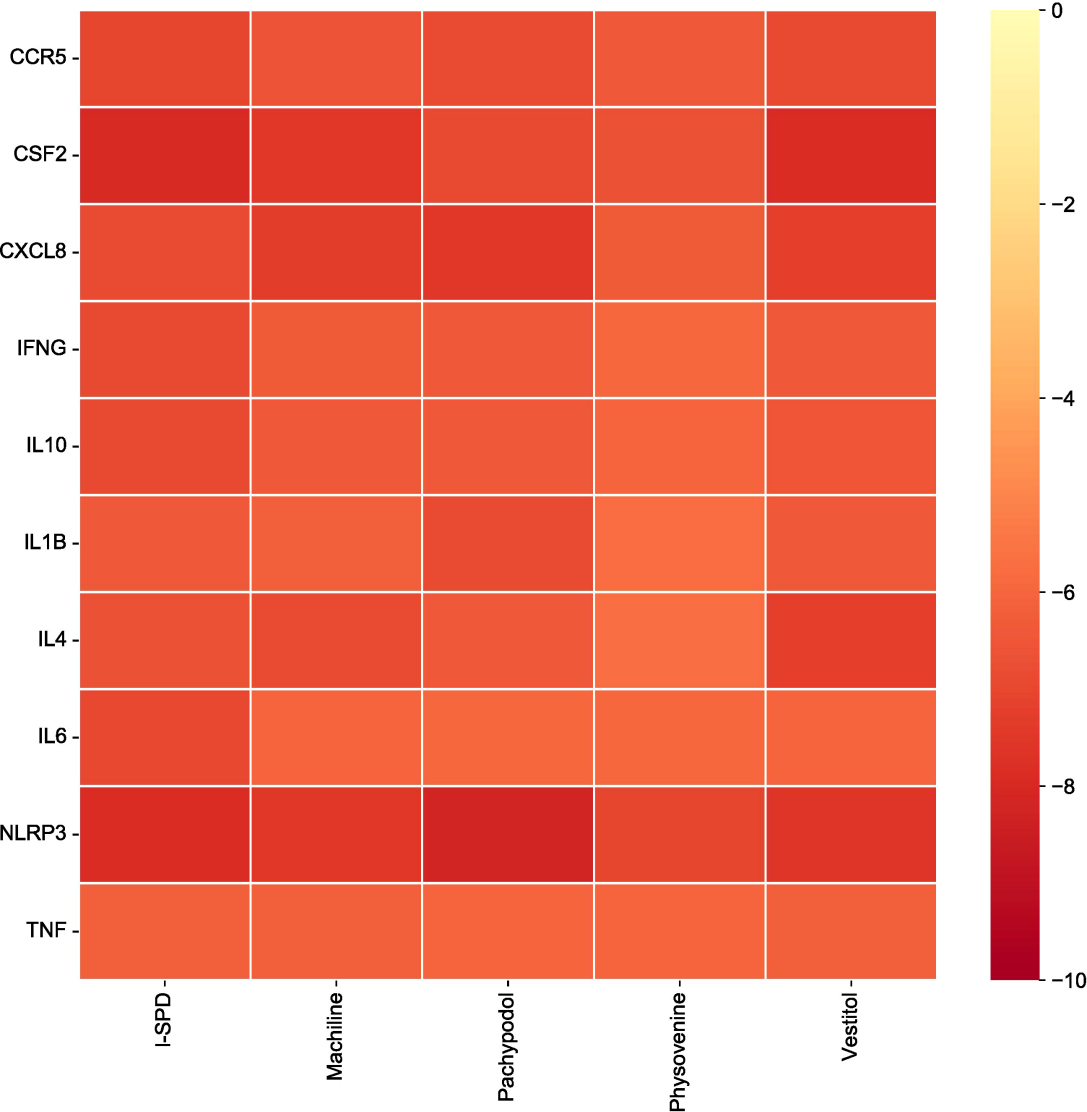
Screening docking results between ligands and receptors.

The 11 core intersection gene targets were selected for molecular docking. The stability of receptor-ligand binding depends on the binding energy. The lower the binding energy of the complex, the more stable the receptor-ligand binding conformation. The results show that the binding of CSF2/I-SPD complex is mainly maintained by hydrogen bonding and hydrophobic interaction. For example, I-SPD can form hydrogen bonding with GLN-43 on CSF2 protein, and also with TYR-71, LEU-42, ILE-104, PRO-105 forms a hydrophobic interaction, shown in **Figure 5A**. The binding of CSF2/Vestitol complex is mainly through hydrophobic interaction, for example, the small molecule Vestitol and PRO-76, LEU-42, TYR-71, ILE-104, PRO-105 on the protein form hydrophobic interaction, shown in **Figure 5B**. In the NLRP3/I-SPD binding complex, the small molecule I-SPD forms hydrogen bonds with GLN-468, SER-470, ALA-72, and also with VAL-197, GLU-473, LEU-472, TYR-476, PHE -419 forms a hydrophobic interaction. In addition, we also observed that I-SPD and ARG-422 form cationic pi conjugation, shown in **Figure 5C**. The binding of NLRP3/Pachypodol suggested that the small molecule Pachypodol forms hydrogen bonds with VAL-197, GLU-200 and GLU-213, and also forms hydrophobic interactions with LEU-199 and PRO-196 on the protein, shown in **Figure 5D**, molecular docking result scores are shown in **Figure 6**.

### Molecular dynamics results

**Figure 7:**
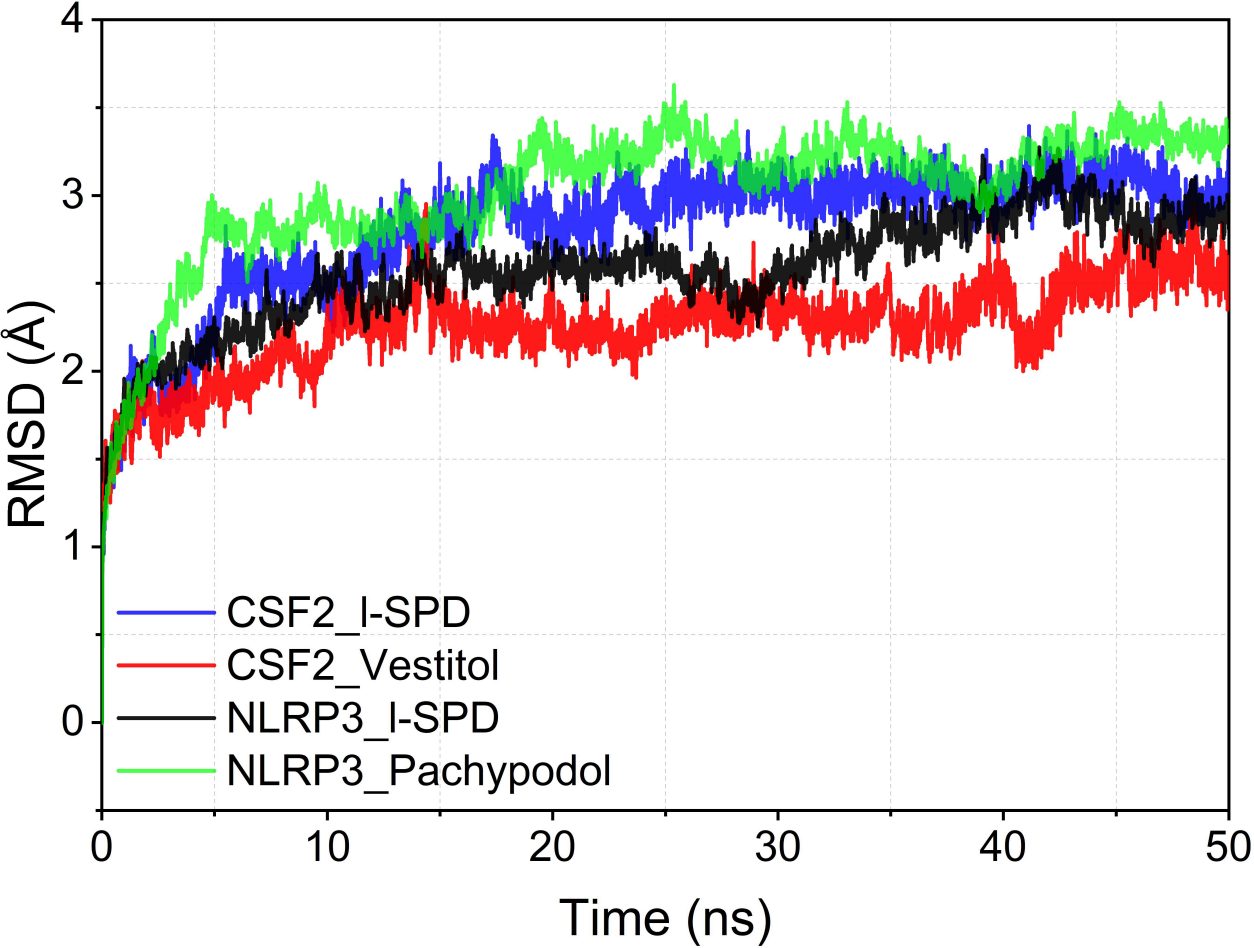
Complex root mean square deviation (RMSD) difference over time. ns, nanosecond.

The root mean square partiality of molecular dynamics simulation is used to reflect the movement process of the complex. The larger the RMSD value of the complex, the more severe the fluctuation and the more intense the movement. On the contrary, the movement is stable. The RMSD of the four systems gradually converged in the first 5 ns of the simulation, and kept stable fluctuations in the subsequent simulations. It is suggested that the motion of the four complexes is stabilized after the combination of the kinetics. In comparison, CSF2/Vestitol (red line) has the lowest RMSD, followed by NLRP3/I-SPD, then CSF2/I-SPD, and finally NLRP3_Pachypodol, indicating that the stability of these complexes is CSF2/I-SPD, CSF2/Vestitol, NLRP3/I-SPD, NLRP3/Pachypodol. However, it is worth emphasizing that the RMSD results of all complexes suggest that small molecules can bind to proteins and maintain a relatively stable state. The results are shown in **Figure 7**.

### Combined free energy calculation results

Based on the trajectory of the molecular dynamics simulation, we calculated the binding energy using the MMGBSA method, which can more accurately reflect the binding mode of small molecules and target proteins. The binding energies of CSF2/I-SPD, CSF2/Vestitol, NLRP3/I-SPD, and NLRP3_Pachypodol complexes were -20.89 ± 1.32 kcal/mol, 27.57 ± 2.78 kcal/mol, -30.52 ± 1.17 kcal/mol, and -21.65 ± 3.36 kcal/mol. The negative values indicate that both molecules have binding affinity for the target protein, and lower value indicate stronger binding. Obviously, our calculations show that these molecules and the corresponding proteins have a certain binding affinity and are very strong. Among them, NLRP3/I-SPD and CSF2/Vestitol have higher binding energies. For the binding energy of the NLRP3/I-SPD complex, the energy decomposition shows that the van der Waals energy is the main contribution energy. For the binding energy of the CSF2/Vestitol complex, the energy decomposition shows that the electrostatic energy is the main contribution energy. The experimental results are shown in **Table 2**.

**Table 2:** Binding free energies and energy components predicted by MM/GBSA (kcal/mol).

System name	CSF2/I-SPD	CSF2/Vestitol	NLRP3/I-SPD	NLRP3/Pachypodol
**Δ*E*_vdw_**	-31.85 ± 0.83	-35.21 ± 1.70	-39.13 ± 4.72	-26.90 ± 1.87
**Δ*E*_elec_**	-74.07 ± 6.98	1.43 ± 2.49	-77.18 ± 10.66	-15.70 ± 5.59
**ΔG_GB_**	88.70 ± 7.47	10.83 ± 2.40	90.77 ± 6.69	24.61 ± 4.35
**ΔG_SA_**	-3.67 ± 0.11	-4.63 ± 0.15	-4.97 ± 0.18	-3.65 ± 0.23
**ΔG_bind_**	-20.89 ± 1.32	27.57 ± 2.78	-30.52 ± 1.17	-21.65 ± 3.36

ΔE_vdW_: van der Waals energy.

ΔE_elec_: electrostatic energy.

ΔG_GB_: electrostatic contribution to solvation.

ΔG_SA_: non-polar contribution to solvation.

ΔG_bind_: binding free energy.

### Hydrogen bond analysis

**Figure 8:**
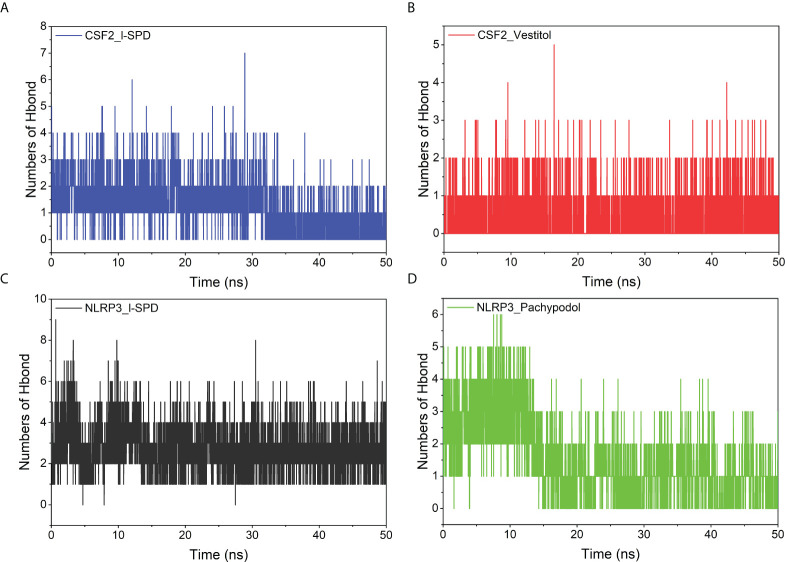
Changes in the number of hydrogen bonds between small molecule ligands and protein receptors in complex system simulations **(A)** CSF2/I-SPD, **(B)** CSF2/Vestitol, **(C)** NLRP3/I-SPD, **(D)** NLRP3/Pachypodol.

Hydrogen bonds are one of the strongest non-covalent binding interactions. The more the number, the better the binding. The results suggest that the number of hydrogen bonds between small molecules and NLRP3 is significantly more than the number of hydrogen bonds with CSF2. Combining the above binding modes, we can see that the number of hydrogen bonds is small. The interaction of molecules and NLRP3 may be dominated by hydrogen bonding, especially the NLRP3/I-SPD complex with the strongest binding energy. The interaction of small molecules with CSF2 may not mainly occur through hydrogen bonding, but through hydrophobic interaction. The results are shown in **Figure 8**.

### The stability of the target protein at the residue level

**Figure 9:**
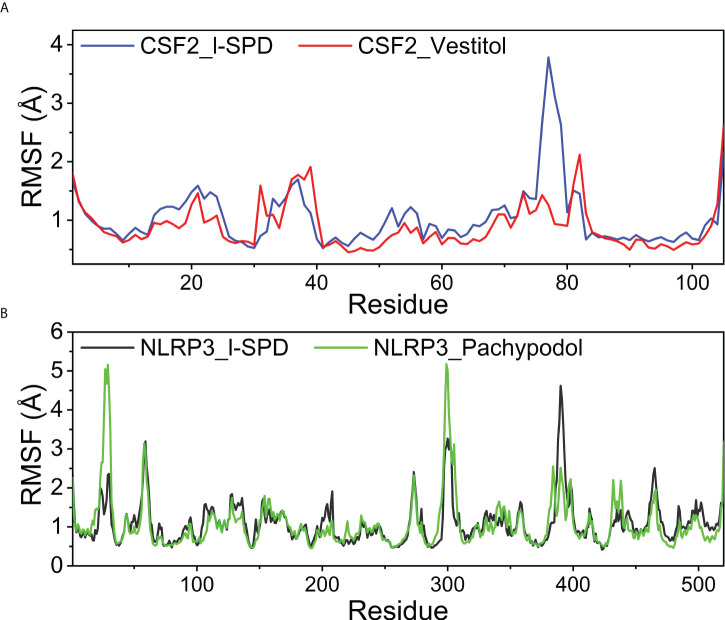
Changes in the stability of protein targets at the residue level **(A)** CSF2/I-SPD and CSF2/Vestitol. **(B)** NLRP3/I-SPD and NLRP3/Pachypodol.

To explore the local fluctuations of macromolecular proteins at the residue level, the vibrations of each residue after compound binding were explored as root mean square fluctuations (RMSF). RMSF can reflect the flexibility of proteins during molecular dynamics simulations. Usually, after the drug binds to the protein, the flexibility of the protein decreases, thereby achieving the effect of stabilizing the protein and exerting the effect of enzymatic activity. The RMSF of the CSF2 and NLRP3 proteins after binding different small molecules is generally low, indicating that the protein as a whole has good rigidity, shown in **Figure 9**. It is worth noting that for CSF2, the decrease in RMSF after the binding of Vestitol small molecule indicates a significant decrease in protein rigidity; however, for NLRP3, the effect of I-SPD and Pachypodol on protein RMSF was not different.

### Analysis of the radius of gyration

**Figure 10:**
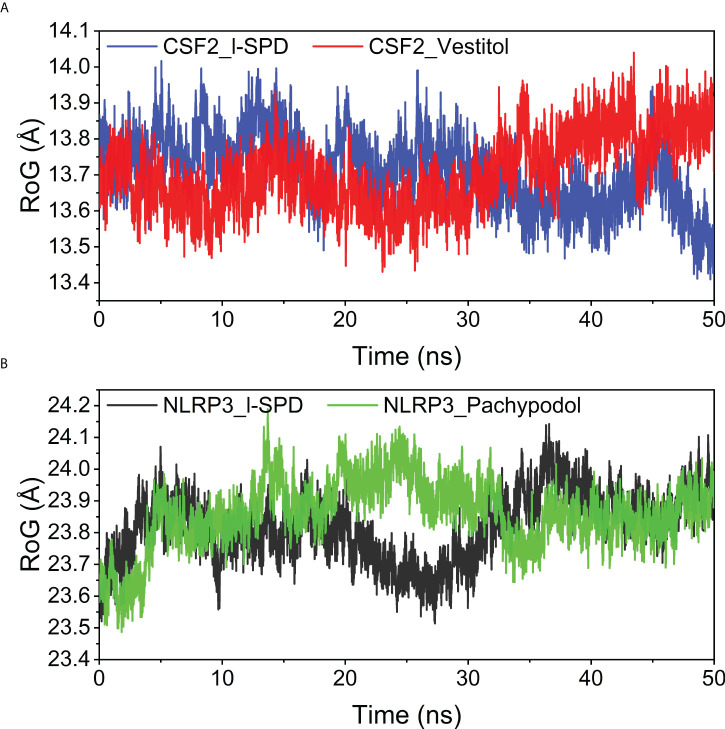
Analysis of protein folding state and overall conformation **(A)** CSF2/I-SPD and CSF2/Vestitol. **(B)** NLRP3/I-SPD and NLRP3/Pachypodol. ns, nanosecond.

The radius of gyration (Rg) reflects the compactness of the embodiment and can reflect the degree of binding of the system. For the CSF2 protein, the Rg after combining two small molecules acts at 13.7 angstroms; for the NLRP3 protein, the compactness after combining the small molecules is about 23.8 angstroms. The overall values are low, implying that the system is denser and more closely combined. It is worth mentioning that the CSF2 protein is smaller, and the Rg of CSF2 is smaller than that of NLRP3, the results are shown in **Figure 10**.

### Analysis of solvent accessible surface area

**Figure 11:**
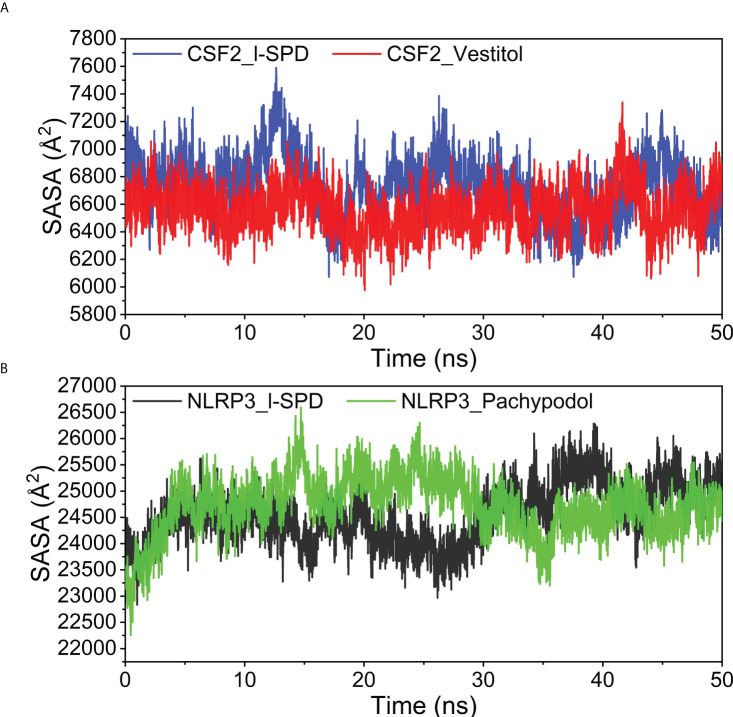
Analysis of Solvent Accessible Surface Area (SASA) **(A)** CSF2/I-SPD and CSF2/Vestitol. **(B)** NLRP3/I-SPD and NLRP3/Pachypodol. ns, nanosecond.

Solvent accessible surface area is calculated as the interface surrounded by solvent. This solvent behaves differently under different conditions and is therefore a useful parameter for studying protein conformational dynamics in a solvent environment. The contact area between the four complexes and water is similar, and the small molecule has little effect on the effect of protein and water, the results are shown in **Figure 11**.

## Discussion

This study explored the pharmacological mechanism of *Xuanfei Baidu granule* (XFBD) in the treatment of COVID-19 by molecular docking and molecular dynamics simulation based on molecular system movement. For the first time, it was found that the important active chemical components I-SPD and Pachypodol in XFBD could reduce the inflammatory response and apoptosis by inhibiting the activation of NLRP3, and reduce the production of inflammatory response. And I-SPD and Vestitol could inhibit the activation and chemotaxis of inflammatory cells through CSF2, prevent the generation of inflammatory storm. Therefore, Vestitol, Pachypodol and I-SPD in XFBD could effectively treat COVID-19 through NLRP3 and CSF2 and reduce the clinical symptoms of patients.

### Bioinformatics analysis of XFBD

Pachypodol, I-SPD and Vestitol in XFBD play a role in treating COVID-19 by acting on NLRP3, CSF2, and relieve the clinical symptoms of SAR-Cov-2 infection.

Pachypodol and I-SPD reduce inflammation and apoptosis by inhibiting the activation of NLRP3, thereby exerting protective effects on the respiratory and nervous systems of patients. Analysis of protein interaction network PPI suggested that NLRP3 was closely related to viral infections and inflammatory responses targets, GO analysis results suggest that NLRP3 is mainly located in extracellular space, KEGG pathway analysis found that NLRP played a role in coronavirus disease COVID-19, influenza A and other pathways. The analysis results suggest that the SARS-CoV 3a protein, as a transmembrane pore-forming viral protein, can activate the NLRP3 inflammasome by forming ion channels in macrophages. At the same time, NLRP3 is found to play a role in pathways such as influenza A, and the inflammasome NLRPS can induce the production of the inflammatory cytokine IL-10 in host cells, resulting in an inflammatory cytokine storm. Inflammatory cytokine storms can cause acute respiratory distress syndrome (ARDS) and acute lung injury (ALI).

Vestitol and I-SPD mainly act on CSF2 to suppress cytokine storm and infiltration of immune cells. CSF2 was closely related to inflammatory targets in PPI. GO analysis results suggest that CSF2 is mainly located in extracellular region. KEGG pathway analysis found that CSF2 played a role in cytokine-cytokine receptor interaction and other pathways. CSF2 can be seen as an attractive mediator. CSF2 is produced as a pro-inflammatory cytokine by many cells, including macrophages, T cells, endothelial cells, and epithelial cells. CSF2 can control the production and differentiation of granulocytes and macrophages, and CSF2 has the effect of promoting tissue inflammation

However, the current bioinformatic analysis results can only predict potential relationships between drugs and gene targets and proteins. Therefore, the use of molecular docking and molecular dynamics in this study can verify the potential relationship of XFBD in the treatment of COVID-19.

### Analysis of molecular docking and molecular dynamics

There is a strong affinity between active ingredient of medicine (such as Pachypodol, I-SPD and Vestitol) and the protein targets (such as NLRP3 and CSF2) through molecular docking tests. Molecular dynamics suggest that they can maintain a very stable binding state, and then play a pharmacological role in the treatment of COVID-19.

I-SPD could stably act on NLRP3 and CSF2, especially NLRP3/I-SPD showed strong stability. Molecular docking showed that the binding energies of small molecules to NLRP3 and CSF2 reached -7.9 and -8.0. Based on the trajectory of the molecular dynamics simulation, we calculated the binding energy using the MMGBSA method, which could more accurately reflect the binding mode of small molecules and target proteins. The binding free energy results showed NLRP3/I-SPD and CSF2/I-SPD were -39.13 ± 4.72 kcal/mol and -31.85 ± 0.83 kcal/mol, for the binding energy of the NLRP3/I-SPD complex, the energy decomposition showed that the van der Waals energy was the main contributing energy. In the molecular dynamics simulation, the RMSDs of NLRP3/I-SPD and CSF2/I-SPD both converged gradually in the first 5 ns of the simulation and preserved stable fluctuations in subsequent simulations, implying that the kinetics of the four complexes are stabilized after binding, and CSF2/I-SPD binding was more stable than NLRP3/I-SPD. NLRP3/I-SPD binding results suggested that small molecule I-SPD forms hydrogen bonds with GLN-468, SER-470, ALA-72, and also formed with VAL-197, GLU-473, LEU-472, TYR-476, PHE-419 Hydrophobic interaction.

The binding of Pachypodol to NLRP3 is relatively stable, molecular docking showed that the binding energies of small molecules to NLRP3 reached -8.2. The binding free energy results show NLRP3/Pachypodol was -26.90 ± 1.87kcal/mol. The number of hydrogen bonds of NLRP3/Pachypodol is relatively stable. The high fluctuation of residues in NLRP3/Pachypodol may be due to the influence of its own multiple peptide chains. NLRP3/Pachypodol binding results suggested that small molecule Pachypodol formed hydrogen bonds with VAL-197, GLU-200, GLU-213, and also formed with LEU-199 and PRO-196 Hydrophobic interaction.

Vestitol combined with CSF2 can form stable complexe, but there were some abnormal fluctuations, which may be due to the influence of the number and angle of binding bonds. molecular docking showed that the binding energies of small molecules to CSF2 reached -7.9. The binding free energy results showed CSF2/Vestitol was -35.21 ± 1.70 kcal/mol, for the binding energy of the CSF2/Vestitol complex, the energy decomposition showed that electrostatic energy was the main contributing energy. We found that RMSF decreased after CSF2 bound to the small molecule Vestitol, suggesting that protein rigidity was significantly decreased. CSF2/Vestitol binding results suggested that PRO-76, LEU-42, TYR-71, ILE-104, PRO-105 on small molecules and proteins form hydrophobic interactions.

We presented the microscopic evolution process of the complex system from the level of small molecules and protein residues through molecular docking and molecular dynamics. Computer simulations visualized the binding states of NLRP3/I-SPD, CSF2/I-SPD, NLRP3/Pachypodol and CSF2/Vestitol. The simulation results showed that the combination of the four complexes can remain relatively stable in the kinetic simulation, thus providing theoretical support for the role of small molecule drugs.

There are certain differences between Xuanfei Baidu granule (XFBD) and traditional single small molecule drugs in the treatment of COVID-19 (Choudhury et al., 2021; Yan et al., 2021). Because Xuanfei Baidu granule (XFBD) as a traditional Chinese medicine compound contains thousands of active small molecules, XFBD can treat diseases through multiple small molecular components acting on multiple disease-related target proteins, while reducing the adverse drug reactions. Therefore, molecular docking and molecular dynamics can be used to more deeply and objectively study the mechanism of action of small molecules in XFBD that coordinate and interact with each other to treat COVID-19. Some studies have used network pharmacology methods to enrich the targets and pathways of traditional Chinese medicines (such as: Lung Cleansing and Detoxifying Decoction (LCDD)) and explore their therapeutic effects on COVID-19 (Xu et al., 2021). This study not only analyzed and drawed on relevant network pharmacology research results, but also used the supercomputer platform to simulate the relationship between small molecule drugs and protein targets through molecular dynamics. For example, molecular dynamics can show the moverment stable between small molecule drugs and protein targets. The root mean square deviation partiality (RMSD)of molecular dynamics simulation can reflect the movement process of the complex.

Therefore, the results of this study could further explain the mechanism of action and related signaling pathways of XFBD in the treatment of COVID-19.

### Pachypodol and I-SPD can reduce inflammation and apoptosis through NLRP3

As an essential component of the innate immune system, the NLRP3 inflammasome is important for antiviral host defense, and its abnormal activation can lead to pathological tissue damage during infection.

The NLRP3 inflammasome is a high molecular weight protein complex composed of the upstream sensor protein NLRP3 and the downstream effector protein caspase-1 (Lamkanfi and Dixit, 2012). When caspase-1 is activated, it promotes the activation of cytokines IL-1β and IL-18 (Mangan et al., 2018), which eventually leads to cell rupture and apoptosis (Liu et al., 2016; Orning et al., 2019; Liu et al., 2020). During COVID-19, the NLRP3 inflammasome is overactivated (Ratajczak et al., 2021), leading to the production of IL-1β/18 and promoting cytokine storm (Lin et al., 2019). Viruses are stimulators of cytokine release syndrome development (Tisoncik et al., 2012). Cytokine storm usually causes patients to express clinical symptoms such as fever, hypotension, and hypoxemia (Shimabukuro-Vornhagen et al., 2018). Elevated levels of IL-1β produced by the NLRP3 inflammasome further activate neutrophils, resulting in increased levels of the neutrophil extracellular traps (NETs) production. High levels of NETs lead to increased clot formation associated with COVID-19 and damage to endothelial and alveolar cells (Zhao et al., 2021b). Activation of NLRP3 requires at least two steps: initiation and activation (Xue et al., 2019). The first step of initiation is activation of the nuclear factor kappa B (NF-κB) signaling pathway (Gritsenko et al., 2020). NF-κB can enhance the transcription of pro-IL-1β, pro-IL-18 and NLRP3 (Afonina et al., 2017). Moreover, the oligomerization of NLRP3 and the assembly of NLRP3 and pro-caspase-1 into the NLRP3 inflammasome (Strowig et al., 2012), which is mainly composed of adenosine triphosphate (ATP) (Karmakar et al., 2016), oxidized mitochondrial DNA (ox-mtDNA)) (Jia et al., 2020), and mitochondrial reactive oxygen species (mtROS) (Zhong et al., 2016) participated in the completion. SARS-CoV-2 can cross the BBB into the central nervous system, directly infect brain tissue, and affect human neural progenitor cells and brain organoids (Zhang et al., 2020).

I-SPD and Pachypodol have the ability to penetrate the blood-brain barrier and inhibit NLRPS3-mediated inflammatory responses in the central nervous system. SARS-CoV-2 invades brain tissue in two ways: the hematogenous pathway and the neuronal retrograde pathway. BBB permeability is increased in patients with neurodegenerative diseases, promoting SARS-CoV-2 neuroinvasion (Zubair et al., 2020). NLRP3 is activated by SARS-CoV-2 in the central nervous system, and high levels of peripheral cytokines (such as IL-1β and IL-6) can directly pass through the BBB or reduce BBB integrity (Mohammadi et al., 2020), inducing peripheral leukocytes and monocytes penetration, impairs immune homeostasis in the brain (Heneka et al., 2013; Yan et al., 2020). At the same time, NLRP3 promotes the aggregation of peptides into pathogenic fibrils and the production of inflammatory cytokines, promotes mitochondrial dysfunction and apoptosis (Freeman and Swartz, 2020), and evolves into neurological lesions.

Therefore, we believe that I-SPD and Pachypodol can reduce the inflammatory response and apoptosis caused by the new coronavirus by acting on NLRP3, thereby exerting a protective effect on the respiratory and nervous systems of patients.

### Vestitol and I-SPD prevent the generation of inflammatory storm and the infiltration of immune cells by inhibiting the overexpression of CSF2

Colony-stimulating factor 2 (CSF2), also known as granulocyte-macrophage colony-stimulating factor (GM-CSF) (Damiani et al., 2020). CSF2 is produced and secreted by many different types of cells, mainly monocytes, macrophages and eosinophils (Hamilton and Anderson, 2004), and normally regulates inflammatory responses and immune activation (Shi et al., 2006).

CSF2 can induce the survival and activation of macrophages and neutrophils, promote the maturation of alveolar macrophages, and play the functions of phagocytosis and killing of viruses (Mehta et al., 2015). The transcription factor PU.1 potentiates the promoting effect of CSF2 on the maturation of alveolar macrophages (Berclaz et al., 2007). Elevated levels of CSF2 in alveolar macrophages stimulate the production of reactive oxygen species (ROS). CSF2 affects the activation and proliferation of immune cells (Hamilton, 2008), and plays an important role in maintaining immune homeostasis in lung tissue (Rösler and Herold, 2016).

CSF2 regulates the Th1 immune response by inducing the production of dendritic cells (Wang et al., 2000; Miller et al., 2002). Interestingly, CSF2 can exert protective effects in humans. CSF2 can regulate the metabolism of vascular collagen (Ponomarev et al., 2007; Li et al., 2015; Shiomi and Usui, 2015), promote the proliferation and migration of vascular endothelial cells, thereby contributing to the process of angiogenesis (Tisato et al., 2013), and induce keratinocyte proliferation and migration, which in turn stimulates wound healing (Szabowski et al., 2000; Barrientos et al., 2008). CSF2 has been shown to protect the lung by restoring barrier function and stimulating epithelial cell proliferation (Huang et al., 2011), and the alveolar epithelium exerts a protective effect against oxidative stress-induced mitochondrial damage (Sturrock et al., 2012). However, when SARS-CoV-2 infected lung tissue, CSF2 was one of the most up-regulated genes in the cells. A cohort study demonstrated a positive correlation between CSF2 and disease severity in COVID-19 patients (Zhao et al., 2021c). High levels of CSF2 are found in the blood of severe COVID-19 patients (Wu and Yang, 2020), so CSF2 is a proxy for excessive inflammation in severe COVID-19 patients (Kluge et al., 2020). When CSF2 is overexpressed in the body, activated monocytes induce T cell death, resulting in lymphopenia, pathological hyperinflammatory immune response, pulmonary fibrosis and severe immune cell infiltration (Xing et al., 1996).

The crucial downstream signaling of CSF2R has been shown to involve JAK2/STAT5 (Lehtonen et al., 2002), ERK (Hansen et al., 2008; Achuthan et al., 2018), NF-κB and the phosphoinositide 3-kinase-AKT pathway (Perugini et al., 2010; Van De Laar et al., 2012). CSF2 is regulated by JAK2, and when activated by phosphorylation, regulates the proper differentiation and maturation of macrophages (Notarangelo and Pessach, 2008), and participates in various intracellular signaling pathways such as STAT5 and MAPK (Hansen et al., 2008). Janus kinase (JAK) activates tyrosine kinase, which then phosphorylates STAT3. Phosphorylated STAT3 activates NF-κB and upregulates the expression of inflammatory cytokines, thereby enhancing inflammation, cell damage and fibrosis (Cao et al., 2022). Macrophages repolarize through the CSF2/CSF2R axis to acquire the M1 phenotype (Ao et al., 2017). Mouse experiments confirmed that CSF2-IRF4 signaling can upregulate MHC class II expression (Van Der Borght et al., 2018). CSF2 enhances the antigen-presenting capacity of macrophages by increasing the expression of MHC-II (Ushach and Zlotnik, 2016). CSF2 upregulates IRF4 expression by enhancing JMJD3 demethylase activity (Yashiro et al., 2018), and activated IRF4 can upregulate CCL17 expression in monocytes/macrophages, mediating the production of inflammation (Achuthan et al., 2016). CSF2 produces airway inflammation by activating airway eosinophils after segmental allergen challenge (Liu et al., 2002). CSF2 induces infiltration and activation of eosinophils in the Th2 network (Nakagome and Nagata, 2011), producing and releasing specific granule proteins *in vitro* (Nagata et al., 1998), ultimately leading to airway pathology. The use of anti-CSF2 receptor monoclonal antibodies to target patients with severe pulmonary disease in COVID-19 can significantly improve clinical symptoms (De Luca et al., 2020; Temesgen et al., 2020).

Therefore, we believe that I-SPD and Vestitol inhibit the overexpression of CSF2 and prevent the generation of inflammatory storm and infiltration of immune cells, preventing mild and common COVID-19 patients from turning into severe ones.

### The mechanisms analysis of Xuanfei Baidu in the treatment of COVID-19

The summary of the mechanisms analysis of *Xuanfei Baidu granule* (XFBD) in the treatment of COVID-19 is shown in **Graphical Abstract**.

## Conclusion

This study revealed the pharmacological mechanism of *Xuanfei Baidu Granule* (XFBD) in the treatment of COVID-19 through molecular docking and molecular dynamics simulation. The results showed that the important active chemical components I-SPD and Pachypodol in *Xuanfei Baidu Granules* (XFBD) can reduce the inflammatory response and apoptosis by inhibiting the activation of NLRP3, and reduce the production of inflammatory response. I-SPD and Vestitol can inhibit the activation and chemotaxis of inflammatory cells through CSF2, preventing the generation of inflammatory storm.

Therefore, Vestitol, Pachypodol and I-SPD in *Xuanfei Baidu Granules* (XFBD) can effectively alleviate the clinical symptoms of COVID-19 patients through NLRP3 and CSF2.

Current molecular docking and molecular dynamics analyses are difficult to quantify. Since the research based on molecular dynamics is still in the stage of simulation analysis, the body function is a continuous and dynamic process. The process of disease occurrence, drug development and efficacy are also dynamic. This study will verify the pharmacological mechanism of Xuanfei Baidu Granules (XFBD) in the treatment of COVID-19, as well as the target and related signaling pathways of active ingredients through cell experiments in the future.

## Data availability statement

The datasets presented in this study can be found in online repositories. The names of the repository/repositories and accession number(s) can be found in the article/**Supplementary Material**.

## Author contributions

LX, JC, XZ, GX, ZY contributed to the conception of the study; JC, XY, SC, MW, CW, HX, YC, DL contributed significantly to analysis and manuscript preparation; JC, HX, YC, RZ, XH, TC, JT, QD performed the data analyses and wrote the manuscript; XZ, GX, JC, ZY helped perform the analysis with constructive discussions. All authors contributed to the article and approved the submitted version.

## Funding

This study was supported by “Sichuan College Students’ innovation and entrepreneurship training program (S202113705049)”.

## Conflict of interest

The authors declare that the research was conducted in the absence of any commercial or financial relationships that could be construed as a potential conflict of interest.

## Publisher’s note

All claims expressed in this article are solely those of the authors and do not necessarily represent those of their affiliated organizations, or those of the publisher, the editors and the reviewers. Any product that may be evaluated in this article, or claim that may be made by its manufacturer, is not guaranteed or endorsed by the publisher.
